# Autoantibodies Targeting G-Protein-Coupled Receptors and RAS-Related Molecules in Post-Acute COVID Vaccination Syndrome: A Retrospective Case Series Study

**DOI:** 10.3390/biomedicines12122852

**Published:** 2024-12-15

**Authors:** Mauro Mantovani, Paolo Bellavite, Serafino Fazio, Giuseppe Di Fede, Marco Tomasi, Daniele Belli, Elisabetta Zanolin

**Affiliations:** 1 Istituto di Medicina Biologica, 20129 Milano, Italy; mauromantovabi72@gmail.com (M.M.); giuseppe.difede@imbio.it (G.D.F.); marco.tomasi@imbiolab.com (M.T.); segreteria@imbiolab.com (D.B.); 2 Independent Researcher, 37121 Verona, Italy; 3 School of Medicine, Federico II University, 80100 Napoli, Italy; 4 Unit of Epidemiology & Statistical Medicine, Department of Diagnostics and Public Health, University of Verona, 37134 Verona, Italy

**Keywords:** post-acute COVID vaccination syndrome, PACVS, COVID-19 vaccine, adverse events, autoimmunity, autoantibodies, ACE2, G protein-coupled receptors, spike protein

## Abstract

**Background/Objectives**: While post-acute COVID-19 syndrome is well known and extensively studied, the post-acute COVID vaccination syndrome (PACVS) is a more recent nosological entity that is poorly defined at the immunopathological level, although it shares many symptoms with the sequelae of viral infections. **Methods**: This single-center retrospective study reports a case series of 17 subjects vaccinated with mRNA or adenoviral vector vaccines who were healthy before vaccination and had never been infected with SARS-CoV-2 but who presented with symptoms similar to PACVS for a median time of 20 months (min 4, max 32). The medical records of all patients referred to our outpatient clinic over a one-year period were retrospectively analyzed. **Results**: In this group, serological tests showed that, in addition to positivity for anti-spike protein antibodies, a high percentage of subjects were positive for antibodies against G protein-coupled receptors and molecules involved in the response to SARS-CoV-2. In a panel of 16 autoantibodies tested, a few were positively associated with some of the symptoms reported by patients: anti-ATR1 with lymphadenopathy and/or tonsillitis; anti-ACE2 with skin symptoms such as ecchymosis, skin oedema, and rash; anti-MAS1 with widespread burning sensation; and anti-STAB1 with skin oedema and rash. Anti-ADRA2A were negatively associated with memory loss and/or mental fog. ACE2 correlated with the serum levels of anti-S antibodies, supporting the hypothesis of an anti-idiotype mechanism in the immunopathogenesis of PACVS. **Conclusions**: This exploratory analysis suggests that the levels of autoantibodies directed against ACE2, and probably also MAS1 and STAB1, may serve as biomarkers for PACVS.

## 1. Introduction

COVID-19 can lead to complications that prolong the course of the disease and make it more difficult to treat (“long COVID” or “post-acute COVID-19 syndrome”, PACS), especially in relation to the severity of the disease [1,2,3,4]. These complications are reported in 10–20% of patients [5,6] and are more common in hospitalized patients [7]. PACS also occurs in vaccinated individuals following breakthrough severe acute respiratory syndrome coronavirus 2 (SARS-CoV-2) infections [8], although with a slightly lower incidence in vaccinated compared to unvaccinated individuals (9.5% vs. 14.2%) [9]. It is becoming clear that even vaccinations against SARS-CoV-2 can be followed by a subacute or chronic pathological condition, the so-called post-vaccination syndrome (PVS) [10] or long post-COVID vaccination syndrome (LPCVS) [11,12,13,14] or eventually Post Acute COVID Vaccination Syndrome (PACVS) [13,15,16]. This condition can be associated with a variety of symptoms that can affect the whole body and even the psyche: general chronic fatigue, muscle and joint pain, dyspnea, numbness of the extremities, orthostatic tachycardia, hypertension, insomnia, anxiety, dizziness, and neurological and neuropsychiatric disorders. Some of these dysautonomia symptoms are also typical of postural orthostatic tachycardia syndrome (POTS) and chronic fatigue syndrome (CFS), both of which have been suggested to have an autoimmune mechanism with the finding of various autoantibodies, including those directed against various types of acetylcholine receptors, against cardiac proteins, β1/2-adrenergic receptors, and M2/3 muscarinic receptors [17]. The distinction between PACVS and PACS is also difficult, because most patients have both a viral infection and vaccination in their clinical history in recent years.

Many signs and symptoms of PACVS, which characterize a disorder of the autonomic system and immunoendocrine responses to vaccination, are similar to those of PACS, suggesting an at least partially common pathogenesis [13,18,19]. Autoimmune mechanisms have also been involved in the adverse effects of vaccination [20,21,22,23,24], but it is unclear what type of antigens and antibodies are involved. The risk of developing autoimmune liver disease [25] or kidney disease [26] is increased after COVID-19 vaccination. In addition, the risk of developing systemic lupus erythematosus is 1.16 times higher in vaccinated individuals compared to controls, and a booster vaccination has been associated with an increased risk of some autoimmune diseases, including alopecia areata, psoriasis, and rheumatoid arthritis [27]. Neurological symptoms such as altered mental status, movement and speech disorders, seizures, and behavioral disturbances have been described as sequelae of autoimmune encephalitis, following both COVID-19 and its vaccines [28]. In the latter literature review, sequelae were reported in 36.7% of COVID-19-associated cases and in 76.9% of vaccine-associated cases. Cutaneous inflammatory symptoms have also been reported as long-term sequelae of COVID-19 vaccines [29,30,31,32,33]. The prevalence of PACVS is estimated to be around 0.02% [15].

Although the mechanisms may significantly differ due to the complex composition of lipid nanoparticles or adenoviral vaccines, the main culprit in determining adverse events is the spike protein, which, in some way simulates the pathogenic action of the virus and triggers dysregulated immune responses, probably with a molecular mimicry mechanism [23,24,34,35,36,37,38,39]. Both SARS-CoV-2 and the spike protein synthesized by vaccines share considerable homology with human proteins or peptides, with an obvious potential to induce autoimmune diseases [5,40,41,42,43]. It is also important to recall the contribution of lipid nanoparticles (LNPs) and other elements characterizing these vaccines, such as polyethylene glycol, cationic lipids (ALC 0315 and SM-102, respectively, for Comirnaty by Pfizer/Biontech and for mRNA 1273 by Moderna), phospholipids, and cholesterol, which can act as adjuvants and therefore promote autoimmune reactions. The ALC-0315 molecules, once penetrated and released into the cytosol, after the disassembly of the LNP envelope, stimulate the secretion of proinflammatory cytokines (such as IL-1 beta and IL-8) and reactive oxygen species, which, in turn, can also have genotoxic effects [44,45].

The aim of this study is to present the case histories of 17 patients who had received one or more doses of vaccine, had no previous history of SARS-CoV-2 infection, and developed symptoms compatible with PACVS after vaccination. Diagnostic investigations included specialized antibody analyses, which are not commonly performed for this type of pathology. After having noted the interesting case of a patient with post-vaccination thrombosis and major anomalies in the autoantibody structure [46], a retrospective analysis of other subsequent cases was performed. Given the small size of the sample, the statistical analyses presented are essentially descriptive. On the other hand, these are cases of post-vaccination syndrome in patients who do not appear to have had COVID-19 and who are negative for anti-nucleocapsid antibodies, rarely observed in our clinical center, and because of the importance of the topic, we have decided to preliminarily communicate the results of this case study.

## 2. Cases and Methods

### 2.1. Cohort of Patients

This study included all consecutive patients with symptoms compatible with PACVS and/or probable autoimmune dysautonomia syndrome that occurred after COVID-19 vaccination and who had not previously been infected with SARS-CoV-2 who attended the outpatient clinic of the IMBIO Medical Institute in Milan (Via Giacinto Gallina, 10, 20129 Milan, +39 02 5830 0445, info@imbio.it). This clinic also has a clinical analysis laboratory and a specialized sampling center (see laboratory analyses).

This is a retrospective observational study on a convenience sample consisting of all patients, with the above characteristics who were examined in 2023. Patients had received one to three doses of the vaccines Comirnaty from BioNTech/Pfizer (P) or Spikevax (mRNA-1273) from Moderna (M), or Vaxzevria from AstraZeneca (AZ). Patients were diagnosed and treated completely independently of the data collection project. None of them had presented any COVID 19-related symptoms, nor were they positive to antigen or molecular swab for SARS-CoV-2 infection, and they stated that they had never previously suffered from the symptoms they presented with, which occurred days or weeks after the anti-COVID-19 vaccination (see Results). All the subjects in our case series had received 1 to 3 doses of anti-COVID 19 vaccines, both adenoviral vector-based and mRNA gene vaccines. Patients were examined on a regular basis at a medical clinic, where a detailed medical history was put together for each patient.

The medical records of eligible patients were reviewed to retrospectively extract relevant clinical and laboratory data, which were coded and sent anonymously to the Department of Diagnostics and Public Health of the University of Verona, Unit of Epidemiology and Medical Statistics, for statistical analysis. The principal investigator (A.M.) and the study research team guaranteed the confidentiality of the participants. Sensitive data of the recruited subjects were processed in accordance with EU Regulation 2016/679 (GDPR) and Legislative Decree 30 June 2003, n. 196 (Code on the protection of personal data). At all stages of the study, only researchers directly involved in the analysis had access to the data, which were stored electronically on a computer with encrypted access.

All patients gave written consent for the use of anonymous data for study purposes. The study was conducted in accordance with the principles of the 1964 Declaration of Helsinki (as amended) and was approved by the IMBIO Institutional Review Board. In addition, this study followed the Strengthening the Reporting of Observational Studies in Epidemiology (STROBE) reporting guidelines.

### 2.2. Laboratory Assays

During the diagnostic activities, autoantibodies associated with classic autoimmune diseases (ANA: antinuclear; ENA: extractable antinuclear; ANCA: anti-neutrophil cytoplasmic; APCA: anti-parietal cell; ASMA: anti-smooth muscle; CMA: anti-cardiac muscle) were measured using commercial kits according to the manufacturer’s instructions (Inova Diagnostics, San Diego, CA, USA). Serum ANA, ANCA, APCA and ASMA were measured by the IF test method (indirect immunofluorescence) using the QUANTALYZER 3000 WERFEN analyzer. ENA and CMA were measured with the chemiluminescence method using with the BIOFLASH WERFEN analyzer (for ENA) and with BIOFLASH (CMA) (Instrumentation Laboratory S.p.A.—Werfen—Milano MI—Italy).

Interleukin-1 beta (IL-1β) and Interleukin-8 (IL-8), collected from saliva according to standard guidelines [47], were measured with a commercial ELISA kit from IBL International GmbH (Flughafenstrasse 52a D-22335 Hamburg, Germany). Quantification of Angiotensin 1–7 (Ang1–7) in serum was performed with competitive inhibition ELISA using a commercial kit marketed and manufactured by Cloud-Clone Corp. (23603 W. Fernhurst Unit 2201, Katy, TX 77494, USA).

To measure total antibodies against SARS-CoV2 IgG and IgM nucleocapsid (ANTI-N) and IgG against SARS-CoV-2 spike protein S-RBD (ANTI-S), the chemiluminescence method is used using the COBAS 602 analyzer (for ANTI-N) produced by ROCHE (Roche Diagnostics (Schweiz) AG—Forrenstrasse 2, 6343 Rotkreuz, Switzerland) and MAGLUMI 4000 Plus (for ANTI-S) produced by SNIBE (Shenzhen New Industries Biomedical Engineering Co., Ltd. Guangdong Province, Shenzhen, Nanshan, 518057—China) [48]. The cut-off values for anti-S and anti-N were 4.3 Binding Antibody Units (BAU)/mL and 1.1 BAU/mL, respectively. A sample equal to or greater than the cutoff was interpreted as positive and a sample showing a value less than the cutoff was interpreted as negative.

In addition, a commercial panel of 16 antibodies was used, including a series of 14 antibodies against GPCR receptors (ATR1: Angiotensin II receptor type 1; ETAR: Endothelin A receptor;ADRA1A: alpha-1A adrenergic receptor; ADRA2A: alpha-2A adrenergic receptor; ADRB1: beta-1 adrenergic receptor; ADRB2: beta-2 adrenergic receptor; CHRM1 to CHRM5: muscarinic cholinergic receptors-1 to -5; MAS1: receptor for angiotensin-(1–7); PAR1: Protease-activated receptor-1, thrombin receptor; CXCR3: Chemokine receptor CXCR3) and against two other receptors related to the renin-angiotensin system (RAS) and potentially involved in COVID-19 and vaccine responses (ACE2: angiotensin converting enzyme 2, the coronavirus spike receptor; STAB1: multifunctional scavenger receptor stabilin-1) [49]. The panel of 16 antibodies was tested from frozen serum using commercial ELISA kits by Prof. Dr. Kai Schulze-Forster and Dr. Harald Heidecke at CellTrend GmbH—ImBio technologiepark, Luckenwalde—according to the manufacturer’s instructions (https://www.celltrend.de/, accessed on 1 June 2024). Briefly, duplicate samples of a 1:100 serum dilution were incubated for 2 h at 4 °C. The ELISA kits were validated according to the Food and Drug Administration’s Guidance for Industry: Bioanalytical Method Validation. Autoantibody concentrations were calculated as arbitrary units (U) by extrapolation from a standard curve of five standards ranging from 2.5 to 40 U/mL. The positivity of the antibody test was established using a cut-off value determined on 77 sex- and age-matched healthy subjects in the same laboratory as previously described [50].

### 2.3. Statistics

Continuous data were described by mean and standard deviations (SD) or median and range if not normally distributed. Normality was assessed using the Shapiro-Wilk test. Qualitative variables were summarized using counts and percentages. As most of the autoantibodies were not normally distributed, the correlation between them was calculated by Spearman’s rho: values of rho between 0–0.25 are considered to show from absent to low association, 0.25–0.50 fair, 0.50–0.75 from moderate to good, 0.75–1 from very good to excellent [51].

The association between the levels of the laboratory variables (coded as binary variables: positive/negative) and the presence/absence of each symptom was analyzed using the Fisher exact test. The non-parametric Fisher test is used in the analysis of contingency tables when the sample sizes are small. The association between the laboratory variables and the presence/absence of each symptom was also analyzed by the Mann–Whitney test, using the laboratory data as continuous values.

Three representative antibodies were chosen to investigate whether the symptoms were more prevalent in subjects with autoantibodies-positive serums than in negative ones, or vice versa. A mixed-effect multilevel logistic regression model was constructed, with any symptom presence as the dependent variable and the positivity/negativity to each of the chosen antibodies as independent variables; a random intercept was included in the model to account for patient-specific likelihood to develop symptoms.

The level of statistical significance was set at *p* < 0.05. The analyses were performed using Stata18.0 (www.stata.com, accessed on 1 September 2024).

## 3. Results

### 3.1. Characteristics of Patients and Symptoms

By analyzing the data of all the patients who met the criteria indicated in the Methods, 17 adults from all over Italy, diagnosed and treated at IMBIO between 2022 and 2023, were included. Table 1 shows the main data of the cases collected.

The patients were 13 women and 4 men with a mean age of 44 years (min 36, max 54) who had received different doses (min 1, max 3) of mRNA (n = 15) or adenoviral vector (n = 2) vaccinations between April 2021 and February 2022. All patients reported that the symptoms had occurred after the vaccinations and that they had not previously suffered from similar illnesses. The onset of symptoms was a few days or at most a few weeks after vaccination (median 10 days, minimum 1 day, maximum 90 days). Twelve patients reported onset of symptoms after the first dose, four after the second dose, and one after the third dose. Thrb14 out of 17 patients reported being in good health prior to vaccination, while three reported having previously suffered from celiac disease (patient n. 4) or West Nile virus fever (patient n. 12), or endometriosis (patient n. 15). The patients we evaluated and considered in our study did not present pathologies or syndromes associated with metabolism (type 2 diabetes and/or clinically diagnosed liver disease or fatty liver) and did not present pathologies associated with cytomegalovirus (CMV) or human papillomavirus infections and/or reactivations. Some had circulating CMV-related IgG but not as a sign of pathology. IgG represent a signal of previous infection but not necessarily of particular clinical conditions related to the infection itself.

The mean interval between the last vaccination and the performance of the autoantibody test was 20.1 months ± 4.5 (min 14, max 32).

Table 2 shows the symptoms extracted from the medical records of each patient, from the highest (left) to the lowest frequency.

Patients reported a minimum of 4 (patient no. 8) and a maximum of 15 (patient no. 5) symptoms, with a mean of 9.1 ± 3.5 (SD) symptoms per patient. The most common symptoms were asthenia, memory or concentration problems and headache, functional changes in the cardiovascular system such as tachycardia and hypertension, other neurological symptoms such as neuralgia, paresthesia, fasciculations, widespread burning sensations, fainting or dizziness, and visual disturbances. Skin alterations, such as edema, rash, bruising, were reported in about one third of patients. It should be noted that the symptoms reported were severe and persistent or recurrent over months or years.

Regarding the two patients with tumors (nos. 2 and 15), it should be noted that patient no. 2 is a 49-year-old man who, after the first and only dose of the vaccine, discovered swollen and painful submandibular lymph nodes. Biopsy and fine needle aspiration were performed 3 months later, and he was diagnosed with non-Hodgkin’s lymphoma. He had no family history and had always been in excellent health. Patient no. 15 is a 54-year-old woman who developed symptoms of chronic fatigue and EBV positivity immediately after the second dose, then worsened after the third dose of the vaccine with the onset of neurological symptoms and was finally diagnosed with cerebral large B-cell lymphoma. She had no family history of blood tumors and was in good health prior to vaccination, except for endometriosis.

### 3.2. Autoantibodies

Table 3 shows the levels of a range of autoantibodies in each patient.

The most frequently altered antibodies in the most patients were those against MAS1, ACE2, CHRM4, ADRA1A and ADRB2, present in 16 (94.1%), 11 (64.7%), 10 (58.8%), 9 (52.9%) and 9 (52.9%) patients respectively. Other commonly altered antibodies were ADRB1, CHRM3 and PAR1, which were present at high levels in 8 out of 17 patients (47.1%). Antinuclear antibodies (ANA), extractable antinuclear antibodies (ENA), anti-neutrophil cytoplasmic antibodies (ANCA), anti-parietal cell antibodies (APCA), anti-smooth muscle antibodies (ASMA) and anti-cardiac muscle antibodies (CMA) were negative in all cases tested (n = 15, missing data for cases no. 1 and 5). In Table 3, some antibodies show quite similar trends between the different patients: for example, those against ADRB1 and ADRB2 are elevated above the normal threshold in the same patients except in one (patient 12), CHRM3 and CHRM4 are elevated in the same patients except in 2 (nos. 4 and 12), in which only those against CHRM4 are elevated).

We have then investigated whether there were any correlations between the antibody levels in our case series (Table 4).

The highest number of correlations was found for ETAR (13 out of 15), ADRB1 and ADRB2 (12 out of 15), STAB1 and CHRM4 (11 out of 15). Excellent correlations between antibody responses were found for ADRB1/ADRB2 (rho = 0.982), CHRM3/CHRM4 (rho = 0.927), ETAR/ADRB1 (rho =0.968), ETAR/ADRB2 (rho = 0.936), ADRB2/CHRM3 (rho = 0.931), ETAR/CHRM3 (rho =0.928). On the other hand, ACE2 was only fairly correlated with ADRA1A (rho = 0.485) and PAR1 was not correlated with any other antibody.

### 3.3. Correlations with Symptoms

To establish whether a particular symptom was correlated with specific autoantibodies, we tested the possible association between the antibodies listed in Table 3 and the presence/absence of each symptom. The Fisher exact test detects the possible presence of a statistically significant difference in the percentage of subjects positive for autoantibodies between those with and without each symptom. The Mann–Whitney test was used to compare the median values of autoantibodies in the two groups of subjects with and without each symptom. The latter is independent of the cut-off value for autoantibody positivity.

The statistically significant results of the Fisher exact test and/or of the Mann–Whitney test are shown in Table 5.

In most comparisons, no statistically significant associations were found between symptoms and autoantibodies. The 6 autoantibodies shown in Table 5—namely ATR1, PAR1, ADRA2A, ACE2, MAS1, and STAB1—correlated with some symptoms according to at least one of the two tests.

Looking at the number of cases that were positive in the antibody test, i.e., those that had antibody doses above the pathological threshold, we observe that ATR1 is positive in two patients (patients n. 1 and n. 14 in Table 3) and both presented symptoms of lymphadenopathy and/or tonsillitis, whereas none of the patients negative for ATR1 presented them (Fisher exact test: *p* = 0.007). The difference is statistically significant with the Fisher test but not with the Mann–Whitney test, probably because the sample size is small, and the test is therefore underpowered. The anti-PAR1 antibody was positive in 8 patients, none of whom had the symptom of lymphadenopathy and/or tonsillitis but was present in 8 out of 13 patients without the symptom (61.5%), suggesting that, in this case. the antibody is significantly associated with the absence of the symptom (Fisher’s exact test: *p* = 0.031).

A special case is the comparison between anti-ADRA2A and memory loss/mental fog, a very common symptom in these patients (15 out of 17): the median of those with the symptom (10.9 U/mL) was significantly different from those without (22.5 U/mL; Mann–Whitney test: *p* = 0.015). The Fisher’s exact test yielded a *p*-value of 0.074 (Table 5).

Considering the anti-ACE2 antibody, it was positive in all 6 patients with the bruising symptom and only in 5 out of 11 (45.4%) of those without the symptom (Fisher exact test: *p* = 0.043). When anti-ACE2 was compared to skin symptoms such as skin oedema and/or rash, the median of those with symptoms (32.3 U/mL) was significantly different from the median of those without symptoms (12.5 U/mL; Mann–Whitney test *p* = 0.014). When comparing anti-ACE2 and skin symptoms such as skin oedema and/or rash, the median of the subjects with symptoms (32.3 U/mL) was significantly different from the median of the subjects without symptoms (12.5 U/mL; Mann–Whitney test *p* = 0.014). Furthermore, 5 patients have this symptom and all of them are positive for ACE2; positivity is also found in 6 out of 12 patients without this symptom (50%): the Fisher exact test shows only a trend towards statistical significance (*p* = 0.102). Having observed these results for anti-ACE2, we also divided the patients according to the criteria of having bruising and/or skin oedema and/or rash (8 patients) or not (9 patients). As shown in Table 5, all 8 patients with these symptoms were positive for anti-ACE2 antibody and only 3/9 (33.3%) of those without symptoms were positive (Fisher exact test: *p* = 0.009). Furthermore, the median ACE2 levels of those with these skin symptoms (26.3 U/mL) and of those without symptoms (8.4 U/mL) were significantly different (Mann–Whitney test: *p* = 0.015).

It is interesting to note the data detected by the anti-MAS1 antibodies, which are above the threshold in all but one patient (Table 3); in addition, the sensation of widespread burning, a serious and common symptom, is present in 12 patients. The median of the autoantibodies in the patients with this symptom was 51.6 U/mL, whereas the median in those without this symptom was 30.9 U/mL, a statistically significant difference (Mann–Whitney test: *p* = 0.009). The Fisher exact test was not significant, probably because almost all patients were positive for the antibody.

The last comparison that showed some significant differences was that of anti-STAB1 in patients with the symptom skin edema and/or rash, which was positive in three out of five patients with symptoms and negative in all those without. Antibody positivity was present in only three patients (n. 4, 11, and 6, see Table 3) and all three had the symptom, whereas none of the patients without symptoms was positive (Fisher exact test: *p* = 0.009). Although the median values showed a difference (17.1 U/mL vs. 45.7 U/mL, in patients without and with symptoms, respectively), the Mann–Whitney test was not statistically significant.

**Figure 1:**
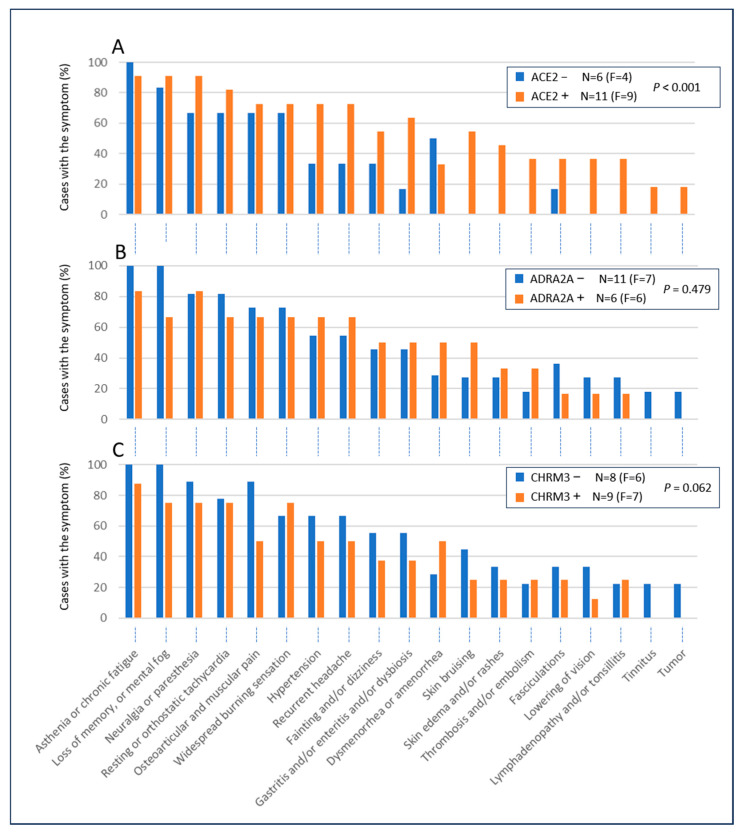
Presence or absence of symptoms in patients negative or positive for anti-ACE2 (panel **A**), anti-ADRA2A (panel **B**) and anti-CHRM3 (panel **C**) antibodies. F is the number of female patients who are negative or positive for the indicated antibodies.

We also wanted to investigate whether the percentage of patients with a specific symptom differed between Ab-positive and Ab-negative patients. Figure 1 shows three antibodies representing three immunologically different situations.

Looking at the symptoms of patients positive for anti-ACE2 antibodies compared to those negative (panel A), we see that the former (orange bars) have a higher percentage of symptomatic cases in 17 of the 19 symptoms considered, apart from two (asthenia and menstrual disorders). Using the multilevel mixed-effect logistic model, we found a statistically significant Odds Ratio (OR) of 4.00 (C.I. 95% = 1.88–8.51, *p* < 0.001), indicating that the ACE2 ab-positive patients have a four-fold risk of having more symptoms with respect to ACE2 ab-negative ones. This trend differs for the other antibodies, two of which are shown in the other panels of Figure 1.

Patients who were positive for anti-ADRA2A compared to negative patients (panel B) had higher percentages for 9 of the 19 symptoms considered. The other 10 symptoms, including asthenia and memory loss (first left blue bars in panel B), have a higher percentage of cases in the ab-negative subjects. In this case, no statistically significant difference between ADRA2A ab-positive and ab-negative patients was found (OR = 1.44, C.I. 95% = 0.52–3.99, *p* = 0.479).

Another interesting case is that of the anti-CHRM3 antibody (panel C): of the 19 symptoms considered, 14 are less frequent in patients who are positive for this antibody than in those who are negative. An OR of 0.41 (C.I. 95% = 0.16–1.04, *p* = 0.062) was obtained, indicating that the CHRM3 ab-positive patients have less than a halved risk of having more symptoms with respect to CHRM3 ab-negative ones. The difference reached only a borderline statistical significance in this case.

We also looked for any association between symptoms, autoantibodies and serum variables (Anti-S, IL-1b, IL-8, and ANG1,7). The only comparisons that yielded statistically significant results, in addition to those already reported above, were a higher amount of anti-S antibodies in subjects with the symptom “widespread burning sensation” compared to those without (median 440 BAU/mL vs. 32.62 BAU/mL respectively, Mann–Whitney test: *p* = 0.036); a higher level of IL-1,b in subjects with “osteoarticular and/or muscular pain” compared to those without (median 347.8 ng/mL vs. 158.9 ng/mL respectively, Mann–Whitney test: *p* = 0.021). Finally, a correlation was found between the levels of anti-S antibodies and of anti-ACE2 antibodies (Spearman’s rho = 0.522, *p* = 0.032).

## 4. Discussion

Although COVID-19 vaccination is effective in protecting against the disease and in particular against its most severe consequences, its biological potency is also reflected in the occurrence of immediate and long-term adverse effects in some individuals. Without considering reactions at the vaccination site, systemic adverse reactions can be interpreted in the context of allergic reactions (e.g., anaphylaxis) or hyper-inflammatory reactions (e.g., hyperthermia), thrombotic disorders or coagulopathies, and autoimmune dysautonomia. Recently, new-onset autoimmune phenomena have been described following COVID-19 vaccination [36], such as vaccine-induced immune thrombotic thrombocytopenia (VITT) syndrome [35,52,53,54], autoimmune neurological diseases [18,55,56], myocarditis and pericarditis [57], cutaneous or systemic vasculitis [29,30], and/or dermatomyositis [31,32,33]. While it is now known that in the case of VITT the pathology is associated with the production of antibodies that recognize platelet factor 4 (PF4) [58], in other diseases the molecular or pathophysiological mechanism of post-acute sequelae of vaccination remains unknown.

Post-vaccination adverse events can also occur in a subacute or chronic form, as well-defined and easily diagnosed diseases (e.g., myocarditis, pericarditis, and Guillain-Barré syndrome) but also in the form of painful and systemic dysautonomia syndromes that do not correspond to well-defined diseases or that share several symptoms of already known diseases but with an obscure pathogenesis, such as fibromyalgia or chronic fatigue syndrome. So far there is no clear definition of this syndrome, although some authors have started to use the acronym Post Acute COVID Vaccination Syndrome (PACVS) [15] or Long Post-COVID Vaccination Syndrome (LPCVS) [14]. In this study we have used the term PACVS, which is the most recent and seems interesting because it recalls the lexical distinction with respect to PACS that occurs after infection with the SARS-CoV-2 virus that is not completely healed. Here, we present data collected in our medical practice over a two-year period, including SARS-CoV-2 naive cases, i.e., cases in which we can assume that the symptoms are due to the vaccine and not to other causes such as viral infection.

To the best of our knowledge, this is the first study in which a high prevalence of autoantibodies against a number of antigens involved in the regulation of the autonomic nervous system and the renin-angiotensin system has been observed in PACVS. The syndrome affecting the patients in our series is not due to an autoimmunity of the type already known, so much so that the classic autoantibodies (ANA, ENA, ANCA: anti-neutrophil cytoplasm; APCA: anti-parietal cell; ASMA: anti-smooth muscle; CMA: anti-cardiac muscle) are all negative in the 15 patients for whom we had the tests available. In a recent paper [59] it was found that in health care workers only 11% of subjects had at least one positivity in a large panel of autoantibodies after anti-COVID-19 vaccination, but those described here were not evaluated. As part of the diagnostic investigations, we introduced the measurement of a panel of antibodies against GPCR receptors and 2 other molecules potentially involved in reactions to COVID-19 and vaccines: ACE2 and STAB1. From this point of view, our study has revealed an additional way of discriminating cases, using the dosage of autoantibodies of the type we identified as most important and correlated with the symptomatic syndrome.

Despite the objectively limited number of cases, we decided to present them and carry out some statistical processing for two reasons: firstly, because ours is a relatively small medical center and does not receive many cases with these characteristics; secondly, because the cases of vaccinated patients who did not also have the disease, perhaps in a mild form, are becoming increasingly rare and therefore increasing the sample size would have required much more time. We believe that the finding of the study, namely the high prevalence of unusual and rarely detected autoantibodies, is novel and deserves to be reported. This work on 17 cases follows a case report communication from our own group [46] in which the immunological study was performed thanks to the panel of 16 antibodies used here, which also served to guide the therapy and follow-up of the patient.

One of the darkest aspects of this type of post-vaccination reaction, which persists over time, is the lack of knowledge about its pathogenesis, which, in turn, makes the therapeutic approach difficult. In fact, all the patients who came to our medical center had been seen by other doctors who had found no therapeutic benefit in any of the drugs they had been given (e.g., antihistamines, corticosteroids, and non-steroidal anti-inflammatory drugs). Furthermore, since many of the symptoms complained of by patients are of a psychological and emotional nature, it is possible that in some cases the chronic post-vaccination syndromes, if not well investigated in the diagnostic process with appropriate instrumental analyses, may be of psychological rather than organic origin. From this point of view, positivity to the autoantibodies described here and statistically associated with disease would be of considerable help for the causality assessment of the adverse reactions following immunization with anti-COVID vaccines.

In our case series, the main causality criteria established by the WHO manual [60] are respected, such as the absence of other causes not related to vaccination, appearing after the injection, a time window compatible with immunological reactions, similarity with other cases of PACVS already described in the literature and biological plausibility, considering the multiform and pleiotropic nature of the possible actions of the spike protein in the body [23,39,61,62,63,64]. All of the patients we observed had complained of the onset of symptoms weeks or at most three months after the administration of the biogenetic anti-COVID-19 vaccines, and these symptoms were still present when they contacted our medical center. A median of 20 months had elapsed between the last vaccination and the antibody test was performed. It is therefore a long-lasting syndrome with some symptoms present in most patients: asthenia or chronic fatigue, memory loss, mental fog, neuralgia or paresthesia, tachycardia, osteoarticular or muscular pain, burning sensations, hypertension, and headache. Other commonly reported symptoms were fainting, dizziness, gastritis or enteritis, skin bruising, and edema or rash. Dysmenorrhea or amenorrhea was reported by more than a third of female patients. These symptoms are consistent with what has been reported by others for PACVS [11,12,13,14,15,16]. Almost all patients in our series experienced severe fatigue and cardiovascular symptoms such as palpitations, tachycardia or hypertension, neurological symptoms such as headache, dizziness and paresthesia, or even psychiatric symptoms such as mental fog, which have been described by others for both PACS and PACVS [19].

Mundorf et al. [15], who studied the case histories of 191 patients with PACVS, found a prevalence of malaise and chronic fatigue in more than 80% of cases and frequent symptoms of cognitive impairment, headache and visual and auditory dysfunction, muscle pain, peripheral nerve dysfunction, paresthesia, and cardiovascular impairment, all symptoms also reported by a large proportion of our patients. Also interesting is the clear prevalence of the female gender in that case history (159 women and 32 men) [15], with which our distributions agree, albeit to a limited extent.

Overall, our study strongly suggests that the analysis of the autoantibody panel we used and especially the dose of anti-ACE2 may have a diagnostic and perhaps predictive utility for this type of post-vaccination pathology. This hypothesis is in agreement with what has recently been reported by other authors [14,15], according to which, PACVS is distinguished from the normal post-vaccination state by altered receptor antibodies (in particular increased ATR1 receptor antibodies and decreased alpha-2B adrenergic receptor antibodies) and increased IL6 and IL-8. In our case series, we also found an increase in ATR1 in 2 out of 17 patients. In addition, we observed increases in many of the other antibodies reported here, of which anti-ACE2 was particularly important, in agreement with a previous report [65]. The latter authors also noted a significant increase of anti-ACE2 from the first to the second dose.

All patients in our series showed one or more positive autoantibodies, but the picture obtained from the immunological panel used was very heterogeneous in the different patients. For most of the antibodies, there was no correlation with symptoms, suggesting that their role is not implicated, or that they are epiphenomena. Only the six autoantibodies reported in Table 5—namely those against ATR1, PAR1, ADRA2A, ACE2, MAS1, and STAB1—correlate positively with some symptoms. The result that appears statistically robust, despite the small sample size, is that anti-ACE2 correlates positively with skin symptoms such as bruising, edema, and rash. This observation is consistent with what has been reported by others, according to which the main skin features in patients with autoimmune vasculitis after COVID-19 vaccination were maculopapular rashes and plaques [30], or rashes and erythema multiforme [66]. Furthermore, from what shown in Figure 1, anti-ACE2 is also positive in the majority of patients who complained of hypertension, headache, visual disturbances, gastritis, or gastroenteritis and in all patients who complained of thrombosis, lymphadenopathy, and/or tonsillitis.

The correlation of anti-ACE2 with skin symptoms and with anti-S antibodies is the most intriguing finding, because the ACE2 molecule/enzyme against which this latter antibody is directed is both the receptor for the spike protein and the enzyme that converts angiotensin 2. This implies, in terms of physio-pathological consequences, that an antibody directed against ACE2 may have functional consequences similar to those of the spike protein itself. By analogy with what has already been suggested for the spike proteins of viruses or vaccines [23,67], the interaction with ACE2 could lead to signaling transduction in inflammatory cells or platelets, with activation and release of inflammatory mediators. Alternatively, the same interaction could lead to the blockade of the enzymatic function and therefore accumulation of angiotensin 2, hypertension and activation of inflammation via the AT1 receptor. It has been observed that ACE2 autoantibody levels are increased in individuals with severe COVID-19 compared to those with mild infection, suggesting a pathogenic role [49,50,68,69,70] and a positive correlation between the levels of antibodies to spike protein (S1-RBD) and autoantibodies to ACE2 has been reported in COVID-19 patients [71]. As for the origin of anti-ACE2, our data, which show a positive correlation with anti-S antibodies, seem to confirm the hypotheses that they are formed by the anti-idiotype mechanism, in agreement with what has been hypothesized by other authors [62,72,73,74]. Some authors have suggested an antigenic cross-reactivity between S1-RBD and its receptor ACE2 [71].

In general, the genesis of PACVS can be attributed to an excessive and, above all, distorted reaction to the inoculated product. Under normal conditions, vaccines induce the synthesis of the spike protein and, consequently, the production of anti-S antibodies, which are expected to exert a neutralizing effect on the virus. However, in certain individuals the production of autoantibodies against epitopes of the spike protein that share similarities with autologous proteins or even against some idiotypes of anti-S antibodies can occur [23,24,37,75]. This phenomenon may be due to a series of factors, such as genetic background (e.g., HLA haplotype [76], MTHFR [77], gender [78,79]), adjuvant effect of lipid nanoparticles [80,81], vaccine impurities [82,83] or batch-dependent heterogeneity [84], or the production of frameshifted recombinant spike proteins [85]. The resulting antibody repertoire can be further amplified through Jerne’s idiotypic network [74,86], producing autoantibodies that can be classified into the following classes: (a) inconsequential molecules without notable function; (b) immunoregulatory antibodies that modulate immune system activity; (c) anti-receptor antibodies causing dysautonomia symptoms on cardiovascular, endocrine, or nervous systems; and (d) autoantibodies prompting inflammatory reactions through immune complex formation, complement activation, cytokine release, and delayed-type cell-mediated immunity. In our study, we have identified certain autoantibodies as being particularly implicated in the onset of symptoms. ACE2 is the major receptor of coronavirus spike protein and STAB1 is a multifunctional scavenger receptor, expressed on macrophages and endothelial cells and is induced during chronic inflammation [87]. ATR1, PAR1, and MAS1 are G-protein-coupled receptors for angiotensin II, thrombin, and angiotensin 1–7, respectively. Interestingly, our data also suggest an inverse relationship between symptoms and the presence of antibodies to two other G-protein-coupled receptors, namely the alpha-2A adrenergic receptor (see Table 5) and the muscarinic cholinergic receptor-3 (see Figure 1), suggesting a potential protective or regulatory role. These observations, derived from our small case series, provide intriguing hypotheses that warrant further investigation. Confirmation through larger epidemiological studies and mechanistic experiments—such as functional assays of these antibodies on cellular and receptor pathways—will be essential to validate these findings and elucidate their implications.

Some authors have observed a significant association between the severity of systemic adverse reactions and higher levels of anti-S antibodies [88]. Other authors have noted that patients with neurological manifestations induced by both infection and vaccination with SARS-CoV-2 were positive for antibodies against ACE2 in the absence of other classic markers of autoimmunity [18]. Independent of COVID-related issues, autoantibodies against ACE2 have been observed to be associated with constrictive vasculopathies [89]. A pro-inflammatory role of anti-ACE2 antibodies is also suggested by the observation that these antibodies are higher in patients with active rheumatoid arthritis than in patients in remission [90]. In theory, when ACE2 receptors become the target of an antibody attack, two potential outcomes can occur. First, if the antibodies exert a blocking effect on the receptor’s carboxypeptidase activity, this could lead to an increase in angiotensin II levels and subsequent activation of the ATR1 receptor. Such activation may result in hypertensive, pro-inflammatory, and endothelial dysfunction consequences [91]. Alternatively, if the antibodies exert a stimulating function on the ACE2 receptor, this could mimic the activity of the spike protein itself and transduce cellular activation signals in endothelia, platelets and leukocytes, contributing to pro-inflammatory cascades [23].

Of the 16 autoantibodies tested, some are strongly correlated with each other (Table 4), and with symptoms (Table 5), while others are independent. Taken together, these results seem to indicate that the post-vaccination syndromes reported in our case series involve multiple autoantibodies and are not due to a single type of immunological alteration. Furthermore, the poor or absent correlation of anti-ACE2 and PAR-1 with other antibodies suggests that the dose of these two antibodies has a different and original diagnostic significance in the syndrome considered here. If the data from our work are confirmed in larger and independent statistics, they would show that the autoimmunity induced by mRNA vaccines against SARS-CoV-2 is very different from those already known and should therefore be studied with appropriate antibody panels.

### Limitations of the Study

The present study has significant limitations, starting from its observational nature and the lack of a control group of healthy subjects, which is impossible in a retrospective study such as our collection of clinical cases. The sample is small, for the reasons mentioned above, and therefore many assumptions that can be drawn from our study cannot be generalized. We included in our retrospective study all patients who consecutively presented with these symptoms, without any selection, but further studies on larger case series are needed to assess whether the high prevalence of autoantibodies of the type we tested is a typical feature of PACVS. It should also be noted that the inclusion of patients in the group defined as having PACVS was mainly symptom-based. Most of the symptoms reported by the patients were not confirmed by neurological examinations, objective measures of nerve function or other cardiovascular clinical investigations. Another limitation of our study is that the positivity of the antibody test was established using a cut-off value that was determined among healthy subjects and COVID-19 patients, not among PACVS patients [50], whose case series are still very small. However, to overcome this limit, the comparison of the Ab levels as continuous variable was performed in subjects with and without the considered symptoms also with Mann–Whitney test (see Table 5), a threshold-independent method. Finally, we could not measure the spike protein in the blood of the vaccinated due to technical limitations related to the complexity and cost of the method, which is not used in our laboratory. A recombinant spike has been found in patients with post-vaccinal myocarditis up to three weeks after vaccination [92] and in vaccinated patients without correlation with symptoms or antibody titers up to 180 days after vaccination [93]. Therefore, we cannot exclude that some of the symptoms reported by patients are due to the spike protein rather than to the effect of autoantibodies. In fact, cardiac beta-adrenergic receptor hyperactivity has also been reported in PASC patients with high plasma levels of spike protein [64].

## 5. Conclusions

This paper underscores the high prevalence of antibodies targeting G-protein-coupled receptors and RAS-related molecules in a series of patients with PACVS, none of which had previous COVID-19 infection. The search for a correlation between symptoms and antibody levels revealed the potential importance of autoantibodies against ACE2, ATR1, PAR1, MAS1, ADRA2A, CHRM3 and STAB1 in the pathogenesis of PACVS, a still poorly defined syndrome. Notably, it was observed that all patients with PACVS report a variety of symptoms, with asthenia, memory loss, neuralgia, orthostatic or resting tachycardia, and muscle pain being the most prevalent. In addition, patients positive for autoantibodies against the spike protein receptor ACE2 exhibit a higher frequency of symptoms such as hypertension, headache, gastritis, skin bruising, edema, or rashes as compared to ACE2-negative patients. If confirmed through systematic studies on larger cohorts, these antibodies could become a valuable biomarker for the diagnosis, clinical evaluation, and management of autoimmune adverse events following COVID-19 vaccination.

## Figures and Tables

**Table 1:** Characteristics of the patients. Underlined in bold are the values above the normal threshold, which is given near the antibody name at the top of each column.

Patient Code	Age	Gender	Doses and Brand of Vaccine *	Date of Last Vaccination	Onset of Symptoms (Days After Vaccine)	Onset of Symptoms (After Dose n.)	Interval (Months) **	Anti-S (4.3 BAU)	Anti-N (1.1 BAU)	IL-1b (143 ng/mL)	IL-8 (110 ng/mL)	ANG1,7 (226 ng/mL)
**1**	48	M	2 P	Jan-22	14	1	18	**40,000**	0.74	46.4	**146.6**	nd
**2**	49	M	1 P	Jan-22	90	1	16	**32.62**	0.12	**323.1**	81	**239**
**3**	44	F	1 P	Nov-21	14	1	19	**27**	1	**234.7**	**152.6**	210
**4**	45	F	3 P	Dec-21	1	2	19	**1764**	0.43	**272.2**	84	**332.9**
**5**	53	M	3 P	Feb-22	1	1	15	**873**	0.07	**557.2**	**228.4**	nd
**6**	37	F	3 P	Jan-22	15	3	14	**446**	0.67	**364.8**	66.8	**229.4**
**7**	40	M	2 P, 1 M	Dec-21	10	2	16	**115**	0.08	46.4	**146.6**	**279.8**
**8**	38	F	1 P	Jun-21	7	1	24	**434**	0.05	**318.3**	79.8	**310.2**
**9**	46	F	2 P	Dec-21	20	1	20	**8.24**	0.92	**158.9**	49	217.4
**10**	37	F	1 AZ	Dec-21	7	1	20	**147**	0.58	**164**	49	167
**11**	50	F	2 P	Dec-21	10	1	21	**103**	0.1	**216.9**	**200**	**292.2**
**12**	42	F	2 P, 1 M	Feb-22	7	1	18	**220.2**	0.09	51.6	**1402**	214.9
**13**	51	F	2 P, 1 M	Feb-22	10	2	21	**297**	0.03	**1052**	**200**	**360.4**
**14**	36	F	1 AZ	Lug-21	7	1	27	**400**	0.88	**574.1**	**144.5**	155.3
**15**	54	F	3 P	Jan-22	10	2	21	**3732**	0.34	**364.4**	**283**	**374.3**
**16**	41	F	1 P	Apr-21	1	1	32	**758**	0.94	30	**303.2**	**365.7**
**17**	38	F	1 P	Feb-22	10	1	22	**1834**	0.45	**595.9**	**488**	**387.5**
**Mean**	44.1				13.8	1.4	20.2	3011.2	0.4	315.9	241.4	275.7
**SD**	6.0				20.3	0.6	4.5	9579.7	0.4	263.7	319.5	77.2
**Median**	44				10	1	20	400	0.43	272.2	146.6	279.8
**Min**	36				1	1	14	8.24	0.03	30	49	155.3
**Max**	54				90	3	32	40,000	1	1052	1402	387.5

* P: Comirnaty/BioNTech/Pfizer, M: Spikevax (mRNA-1273)/Moderna, AZ: Vaxzevria/AstraZeneca. ** Interval time from the date of last vaccination to the date of serological test. BAU: binding antibody units; ANG1,7: Angiotensin 1–7.

**Table 2:** Symptoms reported by each patient, total and percentage of patients with symptoms in the entire case series.

Patient Code	Asthenia or Chronic Fatigue	Loss of Memory or Concentration, Mental Fog	Neuralgia, Paresthesia	Resting or Orthostatic Tachycardia	Osteoarticular and Muscular Pain	Widespread Burning Sensation	Hypertension	Recurrent Headache	Fainting or Dizziness	Gastritis or Enteritis and/or Dysbiosis	Dysmenorrhea or Amenorrhea	Skin Bruising	Skin Edema and/or Rashes	Thrombosis and/or Embolism	Fasciculations	Lowering of Vision	Lymphadenopathy or Tonsillitis	Tinnitus	Tumor
1	**+**	**+**	**+**	**+**		**+**		**+**		**+**					**+**		**+**		
2	**+**	**+**	**+**		**+**							**+**		**+**			**+**		**+**
3	**+**	**+**	**+**	**+**					**+**										
4	**+**	**+**	**+**	**+**	**+**	**+**	**+**	**+**		**+**		**+**	**+**	**+**					
5	**+**	**+**		**+**	**+**	**+**													
6	**+**	**+**	**+**	**+**	**+**	**+**	**+**	**+**	**+**	**+**		**+**	**+**		**+**	**+**		**+**	
7	**+**	**+**	**+**	**+**	**+**	**+**	**+**	**+**		**+**					**+**				
8	**+**	**+**			**+**	**+**													
9	**+**	**+**	**+**	**+**			**+**	**+**	**+**		**+**								
10	**+**	**+**		**+**	**+**	**+**	**+**	**+**	**+**	**+**				**+**		**+**			
11			**+**					**+**			**+**	**+**	**+**						
12	**+**	**+**	**+**	**+**	**+**	**+**	**+**	**+**	**+**	**+**		**+**			**+**			**+**	
13	**+**		**+**		**+**	**+**					**+**								
14	**+**	**+**	**+**	**+**	**+**	**+**	**+**		**+**	**+**		**+**			**+**		**+**		
15	**+**	**+**	**+**	**+**	**+**	**+**	**+**	**+**	**+**		**+**		**+**			**+**	**+**		**+**
16	**+**	**+**	**+**	**+**		**+**	**+**						**+**	**+**					
17	**+**	**+**	**+**	**+**	**+**		**+**	**+**	**+**	**+**	**+**					**+**			
N with symptom	16	15	14	13	12	12	10	10	8	8	5	6	5	4	5	4	4	2	2
% with symptom	94.1	88.2	82.4	76.5	70.6	70.6	58.8	58.8	47.1	47.1	38.5 *	35.3	29.4	23.5	29.4	23.5	23.5	11.8	11.8

* The % of patients with symptom was calculated on females.

**Table 3:** Results of the autoantibody panel used in the serological tests of the case series. Underlined in bold are the values above the normal threshold, which is given near the antibody name at the top of each column.

Patient Code	ATR1 (17 U/mL)	ETAR (17 U/mL)	ADRA1A (11 U/mL)	ADRA2A (15 U/mL)	ADRB1 (15 U/mL)	ADRB2 (14 U/mL)	CHRM1 (9 U/mL)	CHRM2 (9 U/mL)	CHRM3 (10 U/mL)	CHRM4 (10.7 U/mL)	CHRM5 (14.2 U/mL)	ACE2 (9.8 U/mL)	MAS1 (25 U/mL)	PAR1 (4.2 U/mL)	CXCR3 (40 U/mL)	STAB1 (40 U/mL)	Total Positive	% Positive
1	**55.7**	**56.1**	**53.4**	10.7	**70.8**	**83.8**	7.0	6.4	**69.3**	**61.8**	12.2	**36.0**	**52.3**	3.1	11.3	24.0	9	56.3
2	3.8	6.7	6.5	7.4	3.7	4.2	2.3	6.0	5.8	7.4	7.9	**21.0**	**30.9**	4.0	7.1	11.6	2	12.5
3	4.2	6.5	7.5	5.7	5.3	5.0	2.2	3.6	4.0	6.6	6.9	4.7	**28.7**	3.1	9.7	16.5	1	6.3
4	7.4	8.7	11.2	15.0	10.9	9.8	7.5	**9.9**	8.8	**11.8**	13.4	**31.5**	**48.8**	3.5	17.8	**95.1**	6	37.5
5	13.6	14.8	**19.7**	6.7	**27.9**	**34.5**	2.1	5.7	**27.6**	**25.9**	7.2	9.7	**53.1**	**7.2**	13.1	17.2	7	43.8
6	12.6	6.7	7.1	10.1	9.7	9.0	5.1	7.0	4.5	5.9	11.1	**19.3**	**38.4**	**10.3**	9.2	11.6	3	18.8
7	9.3	9.9	8.9	10.3	12.0	12.7	4.1	4.7	8.9	9.6	6.6	7.5	**54.1**	**10.8**	15.4	11.6	2	12.5
8	7.0	8.2	10.6	8.6	11.0	11.6	3.8	6.0	7.5	9.5	9.0	8.2	**48.0**	**4.7**	21.5	15.6	2	12.5
9	8.7	15.6	6.4	**15.1**	**21.1**	**18.2**	8.2	8.3	**15.2**	**15.4**	**15.8**	6.8	**47.0**	3.2	20.1	17.0	7	43.8
10	14.9	**28.9**	**15.2**	**15.3**	**30.0**	**38.1**	5.4	**9.5**	**21.1**	**27.9**	**15.9**	**24.7**	**65.7**	3.8	**83.6**	33.4	12	75.0
11	13.0	15.8	**22.8**	**26.6**	**26.1**	**27.2**	**9.8**	**36.2**	**19.4**	**35.1**	**19.6**	**32.3**	11.0	**55.8**	**128.7**	**125.9**	13	81.3
12	9.8	10.1	9.4	12.1	14.0	**15.8**	3.8	6.0	8.9	**14.5**	11.3	**20.3**	**42.2**	1.5	13.5	24.8	4	25.0
13	13.1	15.1	**12.5**	**18.4**	**26.1**	**29.1**	6.6	**11.6**	**12.7**	**13.8**	13.5	8.4	**51.5**	**5.6**	15.4	22.2	9	56.3
14	**17.3**	**21.3**	**18.8**	**17.3**	**32.7**	**46.2**	**10.2**	**13.9**	**19.0**	**19.7**	10.1	**17.4**	**75.3**	3.8	15.4	32.6	12	75.0
15	7.6	7.8	**11.5**	11.0	9.7	6.1	4.0	4.6	4.3	10.5	7.1	**36.4**	**38.2**	2.2	8.1	10.5	3	18.8
16	11.5	12	**11.2**	12	**19.2**	**33.2**	**9.4**	**11.8**	**12.6**	**18.5**	12.6	**35.7**	**51.8**	**6.4**	**42.3**	**45.7**	12	75.0
17	8.1	10.4	6.4	10.9	12.1	11.7	3.8	**10.2**	7.1	8.7	**24.7**	**15.6**	**44.6**	**5.6**	10.0	12.1	5	31.3
Total positive	2	3	9	5	8	9	3	7	8	10	4	11	16	8	3	3		
% positive	11.8	17.6	52.9	29.4	47.1	52.9	17.6	41.2	47.1	58.8	23.5	64.7	94.1	47.1	17.6	17.6		

**Table 4:** Correlations (Spearman’s rho) between the levels of different autoantibodies. Statistically significant values (*p* < 0.05) are underlined in bold.

	ATR1	ETAR	ADRA1A	ADRA2A	ADRB1	ADRB2	CHRM1	CHRM2	CHRM3	CHRM4	CHRM5	ACE2	MAS1	PAR1	CXCR3	STAB1
**ATR1**	1.000															
**ETAR**	**0.819**	1.000														
**ADRA1A**	**0.708**	**0.620**	1.000													
**ADRA2A**	0.449	**0.645**	0.360	1.000												
**ADRB1**	**0.890**	**0.968**	**0.689**	**0.536**	1.000											
**ADRB2**	**0.897**	**0.936**	**0.683**	**0.485**	**0.982**	1.000										
**CHRM1**	0.474	**0.602**	0.363	**0.791**	**0.501**	**0.514**	1.000									
**CHRM2**	0.445	**0.589**	0.261	**0.767**	**0.504**	**0.494**	**0.778**	1.000								
**CHRM3**	**0.818**	**0.928**	**0.691**	0.460	**0.943**	**0.931**	0.463	0.432	1.000							
**CHRM4**	**0.757**	**0.900**	**0.789**	**0.556**	**0.892**	**0.880**	**0.538**	0.430	**0.927**	1.000						
**CHRM5**	0.304	**0.575**	0.074	**0.689**	0.442	0.380	**0.521**	**0.794**	0.392	0.397	1.000					
**ACE2**	0.248	0.211	**0.485**	0.287	0.139	0.167	0.349	0.270	0.163	0.409	0.238	1.000				
**MAS1**	**0.608**	**0.584**	0.409	0.191	**0.656**	**0.728**	0.288	0.199	**0.624**	**0.483**	0.010	−0.059	1.000			
**PAR1**	0.198	0.052	0.049	−0.011	0.076	0.109	0.115	0.336	0.156	−0.030	0.108	−0.144	0.111	1.000		
**CXCR3**	0.300	**0.561**	0.341	**0.612**	**0.507**	**0.523**	**0.601**	**0.569**	**0.561**	**0.570**	**0.511**	0.012	0.366	0.272	1.000	
**STAB1**	0.474	**0.625**	**0.571**	**0.634**	**0.604**	**0.617**	**0.611**	**0.668**	**0.633**	**0.722**	**0.563**	0.329	0.285	0.003	**0.722**	1.000

**Table 5:** Statistically significant associations between symptoms and specific antibodies according to autoantibody positivity (binary variable, Fisher exact test) and serum level (continuous variable, Mann–Whitney test).

	Symptoms Presence			Antibody Positivity			Antibody in Serum (U/mL)		
Antibody	Type of Symptom Yes/No		N	-	+ (%)	*p* *	Median	Interquartile Range	*p* **
ATR1	Lymphadenopathy or tonsillitis	No	13	13	0	0.007	9.8	8.1–13.0	0.784
		Yes	4	2	2 (50)		12.5	5.7–36.5	
PAR1	Lymphadenopathy or tonsillitis	No	13	5	8 (61.5)	0.031	5.6	3.5–7.2	0.13
		Yes	4	4	0 (100)		3.5	2.7–3.9	
ADRA2A	Loss of memory or mental fog	No	2	0	2 (100)	0.074	22.5	18.4–26.6	0.015
		Yes	15	12	3 (20)		10.9	8.6–15.0	
ACE2	Skin bruising	No	11	6	5 (45.4)	0.043	9.7	7.5–35.7	0.301
		Yes	6	0	6 (100)		20.7	19.3–31.5	
ACE2	Skin edema or rashes	No	12	6	6 (50)	0.102	12.7	7.9–20.7	0.014
		Yes	5	0	5 (100)		32.3	31.5–35.7	
ACE2	Bruising or skin edema or rashes	No	9	6	3 (33.3)	0.009	8.4	7.5–15.6	0.015
		Yes	8	0	8 (100)		26.3	19.8–34.0	
MAS1	Burning sensation	No	5	1	4 (80)	0.294	30.9	28.7–44.6	0.009
		Yes	12	0	12 (100)		51.6	45.1–53.6	
STAB1	Skin edema or rashes	No	12	12	0	0.015	17.1	13.9–24.4	0.501
		Yes	5	2	3 (60)		45.7	11.6–95.1	

* Fisher exact test; ** Mann–Whitney test.

## Data Availability

Summary data supporting reported results can be found by the authors. Patients data are unavailable due to privacy restrictions.
